# Initial and mid-term results of LEO Baby stent-assisted coiling of intracranial aneurysms located in small arteries: A single-center experience with 131 consecutive patients

**DOI:** 10.3389/fneur.2022.990532

**Published:** 2022-09-13

**Authors:** Yunan Shen, Heng Ni, Jingfeng Li, Zhenyu Jia, Yuezhou Cao, Haibin Shi, Linbo Zhao, Sheng Liu

**Affiliations:** Department of Interventional Radiology, The First Affiliated Hospital of Nanjing Medical University, Nanjing, China

**Keywords:** low profile stent, intracranial aneurysms, coiling, embolization, small vessels

## Abstract

**Background and purpose:**

Low-profile intracranial stents such as the LEO Baby stents are considered to be advantageous for the treatment of intracranial aneurysms originating from small arteries. This study aimed to evaluate the initial and mid-term clinical and angiographic results of LEO Baby stents in stent-assisted coiling of intracranial aneurysms with small parent arteries (<2.5 mm).

**Methods:**

We performed a retrospective study to identify 131 patients with aneurysms arising from small parent arteries treated with Leo Baby stent-assisted coiling in a single institution between October 2018 and June 2021. We assessed the immediate and progressive aneurysm occlusion rates, procedure-related complications, and clinical outcomes.

**Results:**

A total of 131 patients with 135 aneurysms were identified, including 65 (48.1%) cases of acutely ruptured aneurysms. Technical success was achieved in all cases (100%). The immediate angiography showed complete occlusion in 111 aneurysms (82.2%), neck remnants in 19 (14.1%), and residual sac in 5 (3.7%). Procedure-related complications occurred in 14 cases (10.3%), including 13 (9.6%) thromboembolic complications and 1 (0.7%) hemorrhagic complication. Six-month follow-up angiography was achieved in 106 (78.5%) aneurysms, which showed complete occlusion in 102 (96.2%) aneurysms, neck remnants in 2 (1.9%), and residual sac in 2 (1.9%). Clinical follow-up was available in all patients with a median duration of 6.8 months, and favorable clinical outcomes (modified Rankin Scale score: 0–2) reached 91.6%. The mortality rate was 4.6%.

**Conclusion:**

Our results demonstrate that stent-assisted coiling of intracranial aneurysms located on small arteries using LEO Baby stents is technically feasible, highly effective, and has midterm durability in aneurysmal occlusion.

## Introduction

In recent years, stent-assisted embolization techniques have played an increasingly important role in the endovascular treatment of wide-necked intracranial aneurysms (1, 2). Stents prevent protrusion of coils into the parent artery, promote proliferation of vascular endothelial cells, and further reduce recanalization and retreatment rates (3). However, wide-necked aneurysms located in distal vessels or small vessels remain challenging due to the difficult navigation of larger (0.021- or 0.027-inch) delivery microcatheters to these small arteries (4–6).

In response to this problem, low-profile stents such as LEO Baby stents were introduced because they can be delivered to small tortuous vessels through 0.0165- or 0.017-inch microcatheters (7, 8). In addition, they are also considered to have a moderate flow-redirecting property due to their high metal coverage (9). The use of this stent has been reported in the literature by several institutions, but little has been reported for its treatment of aneurysms originating from small vessels (<2.5 mm) (10–13). Therefore, we present the largest patient cohort with intracranial aneurysms located in parent arteries <2.5 mm treated with the Leo Baby stent, and examine the feasibility, safety, and therapeutic efficacy. The midterm durability of LEO Baby stent-assisted coiling in this kind of aneurysm was also evaluated.

## Methods

### Patient enrolled

Following institutional review board approval of the study, a retrospective analysis of medical data from our center was conducted to screen patients with aneurysms treated with LEO Baby stents between October 2018 and June 2021. The diameter of the parent artery was defined as the minimum diameter of the segment the stent placed, which was obtained by measuring the post-processed images obtained from the patient's preoperative angiographic results. The exclusion criteria were as follows, (1) patients treated by stenting alone without coil embolization; (2) patients with fusiform aneurysms or patients with blood blister-like aneurysms; (3) patients with aneurysms greater than or equal to 2.5 mm in diameter of the parent-artery; (4) patients with aneurysms of parent-artery have a stenosis; and (5) patients were treated with stent-assisted coiling with more than one stent. Further collation of patient and aneurysm characteristics, clinical findings, radiological follow-up results, and all procedure-related complications obtained from the electronic medical record.

### Endovascular treatment

**Figure 1:**
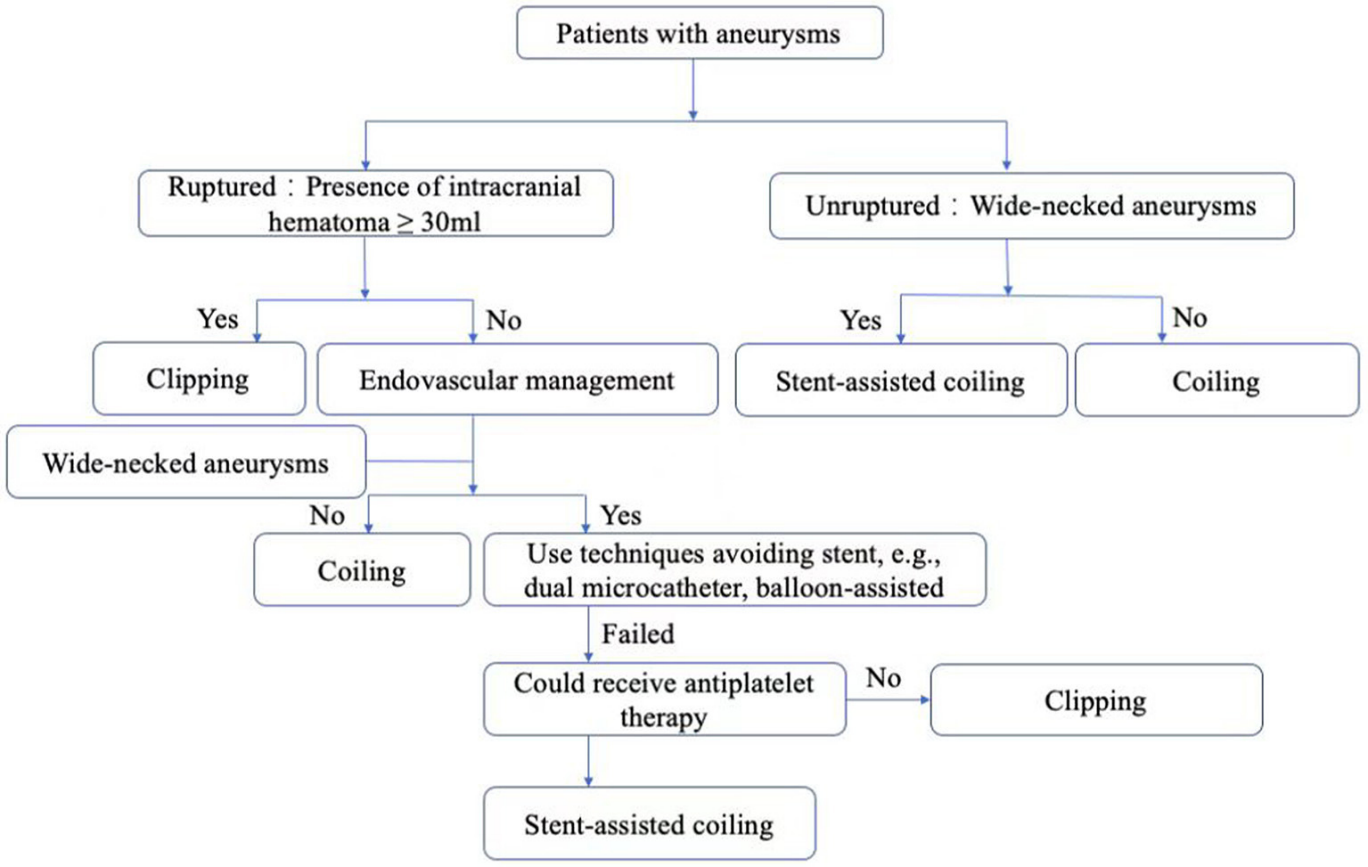
Decision-making chart for treatment options for patients with aneurysms.

All ruptured aneurysms were preferably treated with coiling without stent assistance, and sometimes with balloon assisted or double catheter techniques, except in those cases with an unsatisfactory frame or unstable mass and a low possibility of subsequent evacuation of ICHs or catheterization. For patients with unruptured aneurysms, stent-assisted coiling technique was used in those with wide necks. Leo baby stent was not preferred in aneurysm with an acute angle between parent artery main trunk and involved branch (Figure 1).

All procedures were conducted under general anesthesia. The femoral artery was the primary target puncture site. The standard co-axial technique with a 6-French (Fr) guiding catheter was employed, and systemic heparinization was performed by administering with a loading dose of 70 IU/kg intravenously and maintained with an additional dose of 1,000 IU on an hourly basis to activate the clotting time at 2–2.5 times the patient's baseline level during the procedure. All procedures were performed by two experienced neurointerventional operators (LZ, SL). The Leo Baby stents were delivered *via* either Headway 17 (MicroVention-Terumo) or Echelon-10 (eV3) microcatheters and were deployed using a standard procedure. In all cases, coiling of the aneurysm was performed using semi-jailing technique (In interventions for wide-necked aneurysms, the retractable stent is first partially deployed to cover part of the aneurysm neck, and then fully deployed after completion of coil delivery or satisfactory framing of the aneurysm sac) (14).

### Antiplatelet regimen

According to our institutional dosing criteria, for patients with unruptured aneurysms, dual antiplatelet therapy (100 mg of aspirin and 75 mg of clopidogrel per day) is guaranteed for at least 3 days before treatment. For patients with acutely ruptured aneurysms, tirofiban was injected intravenously with a loading dose (5 μg/kg) immediately after stent insertion and a maintenance dose of 0.1 μg/kg/min for 24 h. Aspirin (100 mg) and clopidogrel (300 mg) were given 20 hours after the procedure, followed by aspirin (100 mg/day) and clopidogrel (75 mg/day) starting at the second day after the procedure. After discharge, all patients continued aspirin (100 mg/day) and clopidogrel (75 mg/day) for 3 months, followed by aspirin alone (100 mg/day) for 1 year. Furthermore, thromboelastogram analysis was used to detect drug sensitivity after 1 week of dual antiplatelet therapy, which assisted us in adjusting the medication regimen.

### Outcome assessment

Technical success is defined as including the successful deployment of the stent and the subsequent successful of the coiling. Safety was considered based on a combination of procedure-related complications, mRS scores at discharge, and 6-month follow-up. Ischemic events include acute thrombosis that occurs intraoperatively and thromboembolism that is confirmed postoperatively. The thromboembolism diagnosis is made when there is a luminal filling defect or when the distal artery does not show. Hemorrhagic events include intraoperative vessel penetration, aneurysm re-rupture, and delayed intracranial hemorrhage. Hemorrhagic complications were diagnosed when there was any sign of contrast leakage due to an aneurysm or vessel rupture during the procedure, or when any immediate or delayed intracranial hemorrhage occurred. Angiography was performed immediately to evaluate the exact degree of aneurysm occlusion after the procedure. The follow-up angiography was routinely performed about 6 months after treatment. All the patients were recommended magnetic resonance angiography or computed tomography angiography 1 year after the first follow-up angiography and at every 2 years thereafter. Angiography was used to analyze the degree of embolization of IA according to the Raymond classification (I, complete embolization; II, residual neck; III, residual aneurysm) (15). A good clinical outcome was defined as an mRS score of 0–2 and an unfavorable outcome as an mRS score of 3–5 according to the mRS score at the 6-month follow-up. In-stent stenosis is defined as the presence of a loss in diameter compared to the initial vessel before treatment, and is considered significant when greater than 50%, otherwise it is non-significant.

## Result

### Patients and aneurysms

A total of 135 intracranial aneurysms in 131 patients were treated with LEO Baby stent-assisted embolization between October 2018 and June 2021. There were 58 male and 73 female patients with a mean age of 59.9 ± 9.8 years (range, 29–85 years). Multiple aneurysms were observed in 43 patients (32.8%). Sixty-four (48.9%) patients were treated for ruptured aneurysms, and two of these patients underwent LEO Baby stent-assisted embolization of another aneurysm at the first subsequent follow-up. All patients with ruptured aneurysms had no abnormalities before the onset of SAH, except one with mRS 3 because of infarction history. The clinical condition of patients with ruptured aneurysms at admission was Hunt and Hess grade 1–2 in 47 (73.4%), and grade 3–4 in 17 (26.6%). Sixty-seven (51.1%) patients were treated for unruptured aneurysms, including 6 patients who had a history of other ruptured aneurysms. Among the patients with unruptured aneurysms, three patients had partial neurological deficits (mRS 3), all of which were sequelae of cerebral infarction. The median size of the aneurysm was 3.60 mm (interquartile range, 2.76–5.10 mm), the mean aneurysm neck was 3.24 ± 1.28 mm, and the median diameter of the parent artery was 1.95 mm (interquartile range, 1.66–2.21 mm), and the smallest diameter was 1.25 mm. These characteristics are described in Tables 1, 2.

**Table 1:** Characteristics of 131 patients.

**Variable**	**Overall**
Age, mean ± SD	59.9 ± 9.8
Female sex (*n*, %)	73 (55.7)
Hypertension (*n*, %)	95 (72.5)
Smoking (*n*, %)	9 (6.9)
Diabetes (*n*, %)	16 (11.6)
Multiplicity of aneurysms (*n*, %)	43 (32.8)
**mRS scores before SAH (** ***n*** **, %)**
0–2	126 (96.2)
≥3	5 (3.8)
**Hunt and Hess Scale (** ***n*** , %)*
I–II	47 (73.4)
III–IV	17 (26.6)

^*^Evaluated in 64 patients with SAH.

SAH, subarachnoid hemorrhage.

**Table 2:** Characteristics of 135 aneurysms.

**Variable**	**Overall**
Aneurysm size (mm), median (IQR)	3.60(2.76, 5.10)
Neck size (mm), mean ± SD	3.24 ± 1.28
Wide (≥4 mm)	35 (25.9)
Narrow (<4 mm)	100 (74.1)
Parent-artery diameter (mm), median (IQR)	1.95 (1.66, 2.21)
**Dome-to-neck ratio**
Favorable (≥2)	12 (8.9)
Unfavorable (<2)	123 (91.1)
**Location**, ***n*** **(%)**
Distal ACA	20 (14.8)
AComA	44 (32.6)
MCA	48 (35.5)
Fetal PComA	15 (11.1)
PCA	4 (3.0)
PICA	2 (1.5)
VBA	2 (1.5)
**Size of aneurysm**, ***n*** **(%)**
Tiny (<3 mm)	32 (23.7)
Small (3–10 mm)	99 (73.3)
Large (10–25 mm)	3 (2.2)
Giant (> 25 mm)	1 (0.7)

SD, standard deviation; IQR, interquartile range; ACA, anterior cerebral artery; AComA, anterior communicating artery; MCA, middle cerebral artery; PComA, posterior communicating artery; PCA, posterior cerebral artery; PICA, posterior inferior cerebellar artery; VBA, vertebrobasilar artery.

### Immediate outcomes and procedural related complications

Stent-assisted coiling was performed successfully in 135 cases (100.0%). In three patients, the LEO Baby stents slipped a short distance proximal to the parent artery during the final retraction of the guidewire, but the stents remained intact over the aneurysm neck without adverse clinical events. Immediate angiography after the procedure showed complete occlusion in 111 aneurysms (82.2%), neck remnants in 19 aneurysms (14.1%), and residual aneurysms in five aneurysms (3.7%). At the pre-discharge visit, 112/131 (85.5%) patients had an mRS of 0–2, and 19/131 (14.5%) patients had an mRS of 3–5.

Fourteen patients (10.7%) developed procedural related complications, including 13 (9.6%) ischemic events, and 1 (0.7%) hemorrhagic event. Among 13 patients with ischemic events, nine were treated for ruptured aneurysms and the other 4 were incidental. Intraoperative acute thrombosis occurred in nine patients. The thrombus resolved after selective treatment with intraoperative mechanical evacuation, arterial infusion of tirofiban, or postoperative intravenous infusion of tirofiban. Six of these patients remained asymptomatic and the remaining three patients developed cerebral infarction. One patient developed contralateral lower extremity weakness postoperatively, and not improved until discharge (mRS 3). One patient presented with postoperative numbness and weakness of contralateral limbs, and recovered the second day after continuous intravenous pumping tirofiban. Another patient was transferred to the ICU in a coma and was discharged with an mRS score of 4. Four patients had postoperative thrombotic events and were immediately treated with intra-arterial thrombolysis after reconfirmation of the diagnosis, and all four were patients with ruptured aneurysms. Two of them had mild headaches at the time of discharge (mRS 1). One patient was discharged with weakness of both lower extremities and speech impairment (mRS 4). Another patient was discharged with unilateral lower extremity muscle strength of 2 (mRS 3).

The patient who had a hemorrhagic complication during the procedure was treated for an unruptured aneurysm located at MCA bifurcation. The immediate angiography after the procedure showed contrast spillage from a small branch of the right middle cerebral artery lower trunk, which was later treated with coil embolization. A repeat angiogram showed occlusion of the hemorrhagic focus. Postoperative CT suggested extensive subarachnoid hemorrhage. After the procedure, the patient was transferred to the ICU for further treatment and accepted the high-voltage oxygen treatment one month later.

### Follow-up outcomes and delayed complications stent related

Clinical follow-up was available in all patients with a median duration of 6.8 months (interquartile range 6.3–8.4 months). Among them, 120 patients (91.6%) had an mRS score of 0–2, three patients (2.3%) had an mRS score of 3, two patients (1.5%) had an mRS score of 5, and six patients (4.6%) had an mRS score of 6. Of the three patients with an mRS score of 3, two had unilateral limb weakness and the other had residual partial cognitive impairment. Of the two patients with an mRS score of 5, one had a cerebral infarction 2 months after the procedure, and he subsequently underwent rehabilitation at a local hospital. A possible reason for this was the absence of regular antiplatelet medication. The other one was the patient with the hemorrhagic complication mentioned above. Six patients with an mRS score of 6 were all with ruptured aneurysms, and five of them had a poor prognosis after treatment because of extensive subarachnoid hemorrhage, another one died 1 month after discharge due to sudden onset of atrial fibrillation. The four patients with ischemic complications who were discharged with an mRS >2 due to an ischemic event all recovered to varying degrees during the follow-up period. The patient who was transferred to the ICU in a coma improved significantly at the time of follow-up, only complaining of occasional mild headache symptoms (mRS 1). Two patients who were left with unilateral limb weakness at discharge, one returned to normal (mRS 0) and the other still had numbness (mRS 1) at the time of follow-up. The patient who had bilateral lower extremity weakness and with an mRS score of 4 at discharge, got improved to mRS 2 at follow-up, only leaving the symptom of speech dysfluency. As to the patient with hemorrhagic complication, he had an mRS score of 5 at 6 months followed up.

**Figure 2:**
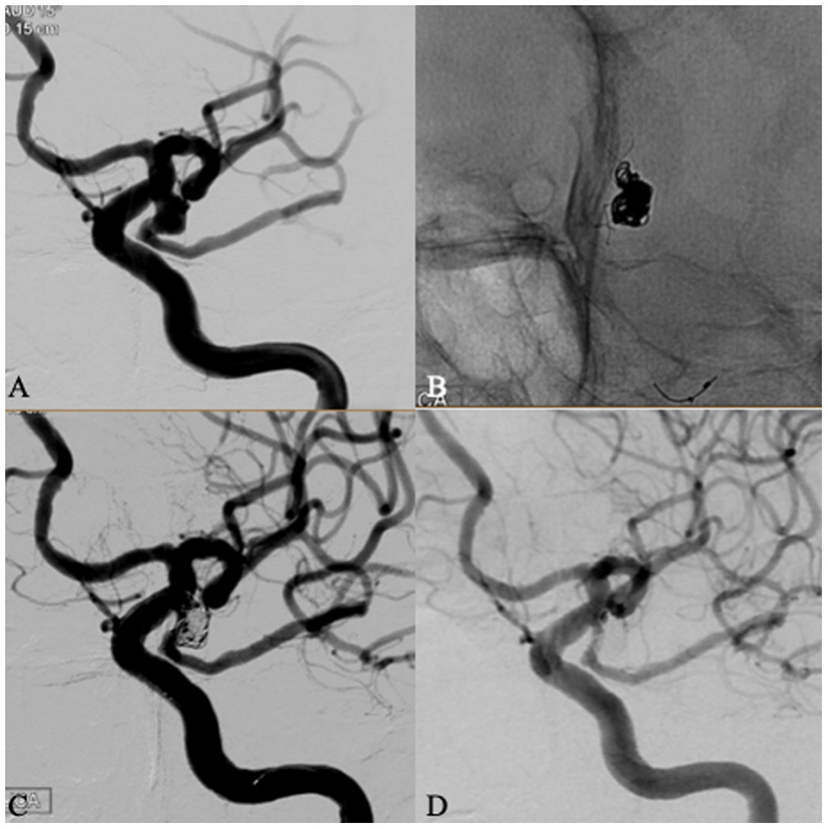
A 64 years old female patient with an incidental fatal posterior communicating (PCom) artery aneurysm. Left internal carotid artery (ICA) angiography showed a PComA aneurysm with wide neck located at fatal posterior communicating artery **(A)**. The aneurysm was coiled assisted by a Leo baby stent deployed in the PCom artery across the aneurysm neck. The proximal end of the stent covered the neck of the aneurysm satisfactorily and was detached in the ICA without touching the opposite wall **(B)**. Immediate angiography after procedure showed a Raymond 3 occlusion **(C)**. The patient was discharged without deficit. Follow-up angiography 6 months later showed a complete occlusion of the aneurysm and patent P-com aneurysm **(D)**.

DSA follow-up (Table 3) was obtained in 106 aneurysms (78.5%), which showed a class 1 occlusion in 102 patients (96.2%), class 2 occlusion in two patients (1.9%), and class 3 occlusion in two patients (1.9%). Follow-up of 20 aneurysms with partial immediate occlusion (Raymond grade 2 or 3) revealed an improvement in Raymond grade (progressive occlusion) in 17 aneurysms (Figure 2). One aneurysm showed recurrence (worsening of Raymond grade), but this patent refused to accept endovascular retreatment. Other patients were followed up by MRA or CTA due to subjective factors (e.g., fear of procedure) or objective factors (geographic or economic reasons), and there were no radiographic results suggestive of aneurysm recurrence.

**Table 3:** Evolution of aneurysm occlusion at first angiographic follow-up.

**Initial treatment**	**Number, *n* (%)**	**First follow-up**, ***n*** **(%)**			
		**Completed follow-up**	**Class 1**	**Class 2**	**Class 3**
Class 1	111 (82.2)	86	86 (100)	/	/
Class 2	19 (14.1)	17	15 (88.2)	1 (5.9)	1 (5.9)
Class 3	5 (3.7)	3	1 (33.3)	1 (33.3)	1 (33.3)
Total	135 (100)	106	102 (96.2)	2 (1.9)	2 (1.9)

**Figure 3:**
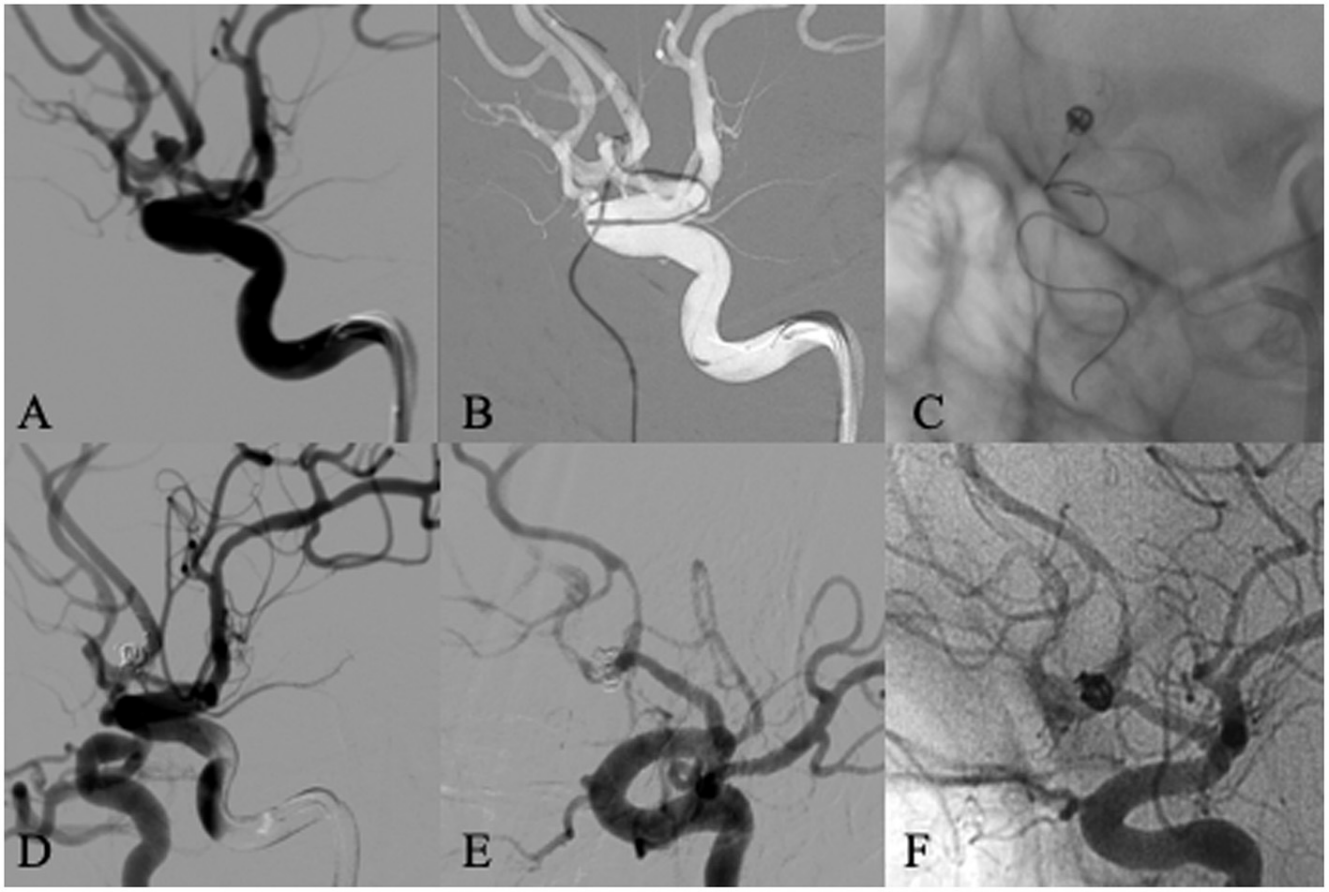
A 59 years old male patient presented with subarachnoid hemorrhage. Left ICA angiogram showed an anterior communicating aneurysm with a daughter sac and a wide neck incorporating both A1/A2 junctions **(A)**. The aneurysm was selected with a microcatheter for embolization from left A1, and a Leo baby stent was deployed from right A1 to left A2, crossing anterior communicating artery and covering the neck of the aneurysm **(B,C)**. The immediate angiogram after embolization showed complete occlusion of the aneurysm and patent anterior cerebral artery **(D)**. Follow-up angiography 6 months later showed in-stent stenosis without any symptom **(E,F)**.

In-stent stenosis (Figure 3) was observed in 11 (10.4%) cases among 106 patients with follow-up angiography, all of which were non-significant (less than 50% of the vessel diameter compared to pre-treatment). And none of these 11 cases were symptomatic. Therefore, statins were given without further endovascular treatment.

## Discussion

In this study, the technical success rate of LEO Baby stent-assisted coiling of aneurysms originating from small vessels was 100%, and the overall procedure-related complication rate was 10.3%. The complete occlusion rate was 82.2% in the immediate postoperative angiographic findings, rising to 96.2% at the first follow-up (median, 6.8 months). Favorable clinical outcomes (mRS score: 0–2) reached 91.6% in the follow-up results. To our knowledge, this is the largest patient cohort with intracranial aneurysms located in small parent arteries (<2.5 mm) treated with the Leo Baby stent.

The stent-assisted coiling (SAC) technique, developed for wide-necked and bifurcated intracranial aneurysms, has greatly expanded the applicability of endovascular therapy (16). The stents provide structural support to the aneurysm neck and prevent protrusion of the spring coil into the aneurysm-carrying artery, allowing for higher density embolization (17). In addition, it provides a support structure for endothelial cell growth to cover the aneurysm neck, providing favorable conditions for a permanent cure of the aneurysm (18). However, for aneurysms located in small vessels, there is a requirement to deliver the stent into the corresponding small vessel, requiring more stringent sizing of the microcatheter. Higher-profile stents represented by Enterprise and Neuroform can only be delivered through microcatheters with a diameter of 0.021-inch or 0.027-inch. Whereas low-profile stents represented by LEO Baby can be delivered through microcatheters with a diameter of 0.0165 or 0.017 inches. Thus, low-profile stents have a greater advantage in terms of microcatheter diameter, especially for aneurysms located at small size vessels. Kim et al. (13) achieved a 100% technical success rate with the placement of low-profile stents (LVIS Jr or Neuroform Atlas) in 66 aneurysm-carrying arteries (<2.0 mm). Earlier, Santillan et al. (19) successfully delivered all LVIS Jr. stents in 35 aneurysms with an I.D. of 0.9–2.5 mm to the target location. And in our current series of 135 cases, the technical success rate was also 100%. This further supports the feasibility of low-profile stents in the treatment of small vessel aneurysms.

In a systematic review and meta-analysis of low-profile stents, the immediate embolization rate of LEO Baby (Raymond class I and II) was 93% (20). In our study, a total of 130 aneurysms (96.3%) had a similar obliteration results, and the complete embolization rate (Raymond class I) reached 82.2%, which is within the range reported in the relevant literature (6–8, 21). Immediate postoperative angiography in the study by Kim et al. (13) showed 57.6% of aneurysms rated as Raymond class I, 19.7% as Raymond class II, and 22.7% as Raymond class III. At the 6-month interim follow-up results, they found a complete embolization rate of 87.9%. In the study by Luecking et al. (11), after a follow-up period with a mean duration of 7.2 months, the rate of complete aneurysm occlusion was observed to increase from 51.9 to 78.9%, and the rate of progressive aneurysm occlusion reached 23.6%. The complete embolization rate in our follow-up results increased to 95.7%, similar to the 96% reported by Machi et al. (22). Meanwhile, we observed progressive aneurysm embolization in 18.3% of cases. The explanation for the presence of progressive embolization could be the low porosity shunting effect of the stent itself. In particular, the Leo Baby stent with high metal coverage has an inherent flow-directing effect, which alters the hemodynamic effect of the aneurysm-carrying artery, shifting the blood flow from the aneurysm sac to the distal parent artery and reducing the hemodynamic pressure leading to aneurysm sac thrombosis (9). Another possible reason is that the stent provides a support structure for the growth of endothelial cells covering the aneurysm neck, thereby inducing neoplastic embolism, which induces neointimal growth and promotes healing of the aneurysm neck, leading to the progressive formation of intracystic thrombus (18).

Procedure-related complications remain a technical limitation of stent-assisted embolization (Table 4). In a previous study on the use of high-profile stents in small vessels, Chung et al. (21) reported 9.7% thromboembolic events without hemorrhagic events in 31 small vessels (<2 mm) using enterprise stents. In a study of aneurysms with a parent vessel diameter of 1.2–2 mm, Kuhn et al. (5) found 13.6% of thromboembolic events and 2.3% of hemorrhagic events. In a recent study on the use of low-profile stents in small vessels, Kim et al. (13) showed 12.1% thrombotic events and 3.0% hemorrhagic events in 66 small vessels (<2 mm) using the LVIS Jr or Neuroform Atlas stent. In another study, Santillan et al. (19) had 11.4% thrombotic events and 2.8% hemorrhagic events in a case series of 35 small vessels (<2.5 mm) using LVIS Jr. We reported a total of 14 (10.3%) complications, which included 13 (9.6%) ischemic events and 1 (0.7%) hemorrhagic event. And these procedure-related complications resulted in a permanent morbidity rate of 3.8% (*n* = 5). It is important to note that 9 of these 13 patients who had an ischemic event were treated for ruptured aneurysms. Xue et al. reported an ischemic complication rate of 7.5% (3/40) in a study using LVIS stent-assisted embolization to treat ruptured middle cerebral aneurysms (24). In a study of acute treatment of ruptured aneurysms using Atlas stents, Russo et al. reported 22 cases of acute thrombosis in 61 patients, with an incidence of 36.1% (25). This is contrary to our conventional belief that laser stents have a lower incidence of thrombosis compared to braided stents. In our series, a total of nine ischemic complications occurred in 64 patients with ruptured aneurysms, with an incidence of 14.1%. Except for the different type of stent used and size of parent arteries, another possible reason for this difference of thrombosis events incidence between each study is that the antiplatelet strategies are various in acute stage. It has been reported that the incidence of thrombotic complications may be higher in patients with SAH due to a hypercoagulable state and insufficient anti-plantelet (24–28). In line with this, the incidence of thrombotic events was slightly higher in patients with ruptured aneurysms (14.1%, 9/64) compared with unruptured aneurysms (6.0%, 4/67) in this study, even tirofiban was used in all ruptured aneurysms with a loading dose and a maintenance dose immediately after stent insertion.

**Table 4:** Comparison of clinical complications with previous studies of stent-assisted embolization of aneurysms located in small vessels.

**Study [Ref.]**	**Stent**	**IAs,** ***n***	**SAH,** ***n***	**PAD,** ** mm**	**Peri-procedural complications, *n* (%)**	**Thromboembolic events, *n* (%)**	**Hemorrhagic events, *n* (%)**	**In-stent stenosis, *n* (%)**	**Procedure-related morbidity, *n* (%)**
Chung et al. (21)	Enterprise	31	16	<2.0	3 (9.7)	3 (9.7)	0	/	/
Kuhn et al. (5)	Neuroform/ enterprise	44	1	≤ 2.0	7 (15.9)	6 (13.6)	1 (2.3)	0	/
Kim et al. (13)	LVIS Jr/Atlas	66	0	≤ 2.0	10 (15.2)	8 (12.1)	2 (3.0)	0	3 (4.5)
Santillan et al. (19)	LVIS Jr	35	10	<2.5	5 (14.3)	4 (11.4)	1 (2.8)	1 (2.8)	1 (2.9)
Alghamdi et al. (6)	LVIS Jr	43	3	<3.5	3 (7.5)	1 (2.5)	2 (5.0)	7 (17.5)	2 (5.0)
Wang et al., (23)	LVIS	22	5	<2.5	1 (4.5)	0	1 (4.5)	1 (4.5)	0
Current study	LEO Baby	135	65	<2.5	14 (10.7)	13 (9.6)	1 (0.7)	11 (10.4)	0

In-stent stenosis is a major concern especially when the stent is placed in small vessels. The incidence rate of in-stent stenosis in laser-carving stents, represented by Enterprise and Neuroform, ranged from 2.5 to 5.8% (29, 30). But in-stent stenosis seems more common in braid stents. Cho et al. (31) reported that the rate of delayed in-stent stenosis was high (86.7%) in stent-assisted coiling using LVIS or LVIS Jr. Of these cases, 69.2% were mild (<33% narrowing relative to non-stented parent artery) and the remaining 30.8% were moderate (33–67%). In previous studies on the LEO Baby stents, Aydin et al. (10) reported incidental in-stent stenosis in 12 patients (15.6%) during a mean follow-up period of 7.2 months, and Luecking et al. (11) reported a total of 2 patients (3.5%) who developed intimal hyperplasia at follow-up. The possible explanation for this relatively high rate of stenosis in braid stents is that the high metallic coverage of this kind of stents may stimulate endothelial proliferation. Alghamdi et al. (6) found that non-significant intrastent stenosis occurred in 17.5 % of midterm followed-up cases with the LVIS Jr stent in small vessels. We observed in-stent stenosis in 10.4% of patients during the 6-month follow-up, which is less than most of those reported with braid stents.

The current study has some limitations. First, this is a retrospective study based on a single-center experience, so some selection bias inevitably exists in the observed samples. Second, the lack of comparison with other low-profile stents. Finally, there is a lack of long-term follow-up, and we will continue to improve the data and further supplement the long-term follow-up results in the future.

## Conclusion

This study confirms that adjuvant embolization therapy with LEO Baby stents for intracranial aneurysms originating from parent arteries smaller than 2.5 mm is feasible and relatively safe, with good aneurysm occlusion rates. Complication rates were acceptable compared with the results of previous SAC studies.

## Data availability statement

The raw data supporting the conclusions of this article will be made available by the authors, without undue reservation.

## Ethics statement

The studies involving human participants were reviewed and approved by the ethical standards of the Institutional Research Committee of Jiangsu Province Hospital, the First Affiliated Hospital of Nanjing Medical University (IRB number: 2020-SR-086). Written informed consent for participation was not required for this study in accordance with the national legislation and the institutional requirements. The Ethics Committee waived the requirement of written informed consent for participation.

## Author contributions

YS and HN analyzed the data and drafted the manuscript. LZ designed the study and helped to revise this manuscript. SL conceived the study and made final approval of this manuscript. JL, ZJ, YC, and HS helped to perform the analysis with constructive discussions. All authors contributed to this article and approved the submitted version.

## Conflict of interest

The authors declare that the research was conducted in the absence of any commercial or financial relationships that could be construed as a potential conflict of interest.

## Publisher's note

All claims expressed in this article are solely those of the authors and do not necessarily represent those of their affiliated organizations, or those of the publisher, the editors and the reviewers. Any product that may be evaluated in this article, or claim that may be made by its manufacturer, is not guaranteed or endorsed by the publisher.
